# Deep ancestry of mammalian X chromosome revealed by comparison with the basal tetrapod *Xenopus tropicalis*

**DOI:** 10.1186/1471-2164-13-315

**Published:** 2012-07-16

**Authors:** Jaroslav Mácha, Radka Teichmanová, Amy K Sater, Dan E Wells, Tereza Tlapáková, Lyle B Zimmerman, Vladimír Krylov

**Affiliations:** 1 Department of Cell Biology, Faculty of Science, Charles University in Prague, Vinicna 7, Prague 2, Czech Republic; 2 Department of Biology and Biochemistry, University of Houston, Houston, TX, 77204-5001, USA; 3 Division of Developmental Biology, MRC-National Institute for Medical Research, Mill Hill, London, NW7 1AA, UK

**Keywords:** X chromosome, Evolution, *Xenopus*, Synteny, Genome, TAG

## Abstract

**Background:**

The X and Y sex chromosomes are conspicuous features of placental mammal genomes. Mammalian sex chromosomes arose from an ordinary pair of autosomes after the proto-Y acquired a male-determining gene and degenerated due to suppression of X-Y recombination. Analysis of earlier steps in X chromosome evolution has been hampered by the long interval between the origins of teleost and amniote lineages as well as scarcity of X chromosome orthologs in incomplete avian genome assemblies.

**Results:**

This study clarifies the genesis and remodelling of the Eutherian X chromosome by using a combination of sequence analysis, meiotic map information, and cytogenetic localization to compare amniote genome organization with that of the amphibian *Xenopus tropicalis.* Nearly all orthologs of human X genes localize to *X. tropicalis* chromosomes 2 and 8, consistent with an ancestral X-conserved region and a single X-added region precursor. This finding contradicts a previous hypothesis of three evolutionary strata in this region. Homologies between human, opossum, chicken and frog chromosomes suggest a single X-added region predecessor in therian mammals, corresponding to opossum chromosomes 4 and 7. A more ancient X-added ancestral region, currently extant as a major part of chicken chromosome 1, is likely to have been present in the progenitor of synapsids and sauropsids. Analysis of X chromosome gene content emphasizes conservation of single protein coding genes and the role of tandem arrays in formation of novel genes.

**Conclusions:**

Chromosomal regions orthologous to Therian X chromosomes have been located in the genome of the frog *X. tropicalis*. These X chromosome ancestral components experienced a series of fusion and breakage events to give rise to avian autosomes and mammalian sex chromosomes. The early branching tetrapod *X. tropicalis*’ simple diploid genome and robust synteny to amniotes greatly enhances studies of vertebrate chromosome evolution.

## Background

Due to suppression of X-Y recombination, the eutherian X chromosome has not undergone major reorganization for over 100 million years and retains an ancestral state [1-3]. Our ability to identify the chromosomal components that gave rise to the X chromosome prior to the mammalian radiation has been limited both by the incomplete state of avian genome assemblies, and by the ancestral teleost whole-genome duplication and subsequent chromosome reshuffling that occurred during the long (>400MY) period since divergence of amniote and fish lineages [4].

Within *Theria*, the human X chromosome long arm and a proximal portion of the short arm correspond to genes on the marsupial X chromosome. This domain, the X-conserved region (XCR), is shared by sex chromosomes of all live-bearing mammals. In contrast, the remainder of the human X short arm, the X-added region (XAR), is autosomal in marsupials [5], and translocated to eutherian sex chromosomes between the divergence of marsupials and placental mammals ~148 million years ago and the eutherian radiation ~100 million years ago [6]. The most basal extant mammal, the egg-laying monotreme platypus, has five pairs of X and Y chromosomes, but these show no homology to the human X. Rather, platypus autosome 6 shares synteny with the entire therian XCR [7,8] including the SOX3 gene from which the testis-determining gene SRY evolved, consistent with this part of the genome being the progenitor of X and Y [9]. The XAR maps to platypus autosomes 15q and 18p.

Broader comparisons within amniotes show that the human XAR is co-linear with a region on chicken chromosome 1, with much of XCR syntenic to the short arm of chicken chromosome 4 [10]. Controversially, another analysis detected synteny between two XCR regions and chicken chromosome 12 plus several microchromosomes, suggesting a third building block in genesis of the human X chromosome [11]. However, subsequent studies argue that putative X orthologs on chicken chromosome 12 and microchromosomes are actually paralogs, and true orthologs of many genes, especially at the border between XCR and XAR, are missing from the current chicken genome assembly [12].

Since the chicken genome assembly remains incomplete, and the duplicated genomes of teleosts have experienced frequent linkage disruptions [13] fragmenting their X chromosome orthology, a different outgroup is required to elucidate tetrapod chromosomal evolution. Recently, the amphibian *Xenopus tropicalis* has been the subject of a genome sequence assembly [14] and a meiotic linkage map [15]. The genome of *X. tropicalis*, unlike those of teleost fish and other *Xenopus* frogs, displays a canonical diploid vertebrate organization, preserving a high degree of synteny to amniote genomes [14]. The present study uses the *X. tropicalis* genome assembly and linkage map in combination with cytogenetic localization to clarify the deep evolutionary origin of the mammalian X chromosome.

## Results and discussion

### Homologies between human X and *X. tropicalis* chromosomes

**Figure 1:**
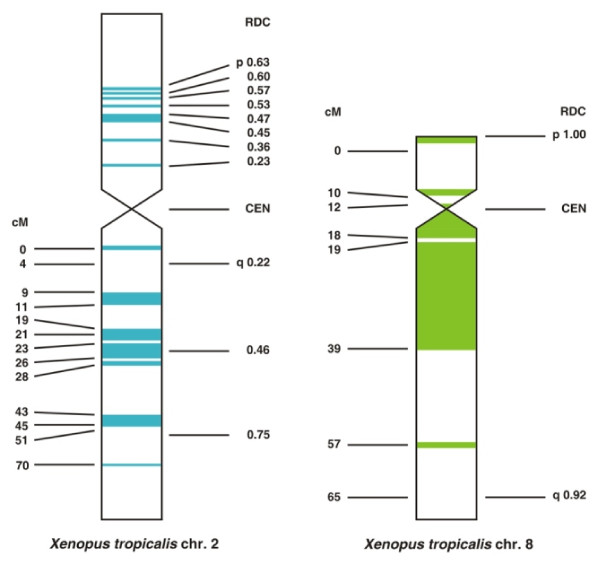
**Positions of scaffolds containing orthologs of human X chromosome genes in *Xenopus tropicalis *chromosomes. ** Of 454 amphibian orthologs of human X-borne genes identified in this study, 442 (97%) localized to *X. tropicalis * chromosomes 2 and 8. Mosaic distribution of X orthologs (blue = XAR; green = XCR) suggests internal rearrangements after chromosomal fusions. cM – centimorgans, rdc - relative distance from centromere, cen – centromere.

We identified putative orthologs of human X chromosome genes in the *X. tropicalis* genome assembly, and obtained the chromosomal locations of 454 of these in two ways. Many X ortholog-containing sequence scaffolds could be directly assigned to linkage groups/chromosomes using the meiotic map. Cytogenetic locations of a subset of these genes, as well as X orthologs from scaffolds not represented on the meiotic map, were also determined by fluorescence in situ hybridization (FISH). In total, 442 (97%) of these X orthologs were found on chromosomes 2 and 8 (Additional file 1); the remaining 12 orthologs are scattered throughout other *X. tropicalis* chromosomes. Intriguingly, many of the scaffolds that had not been localized by genetic mapping were placed by FISH on the short arm of chromosome 2, which is known to be missing from the published meiotic linkage map (Additional file 2) [15]. The known positions of scaffolds containing human X orthologs are displayed in Figure 1.

**Figure 2:**
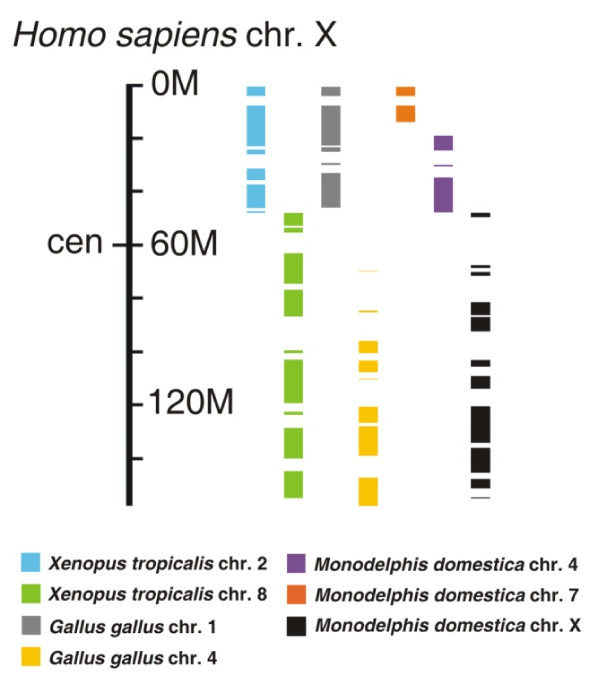
**Regions of homology between human X and *Xenopus tropicalis, Gallus gallus *and *Monodelphis domestica *chromosomes. ** Only gene blocks larger than 0.7 Mbp are shown. Data are from Additional file 1 and the Comparative Genomic display of the Ensembl database [18]. cen – centromere, chr. - chromosome.

On *X. tropicalis* chromosomes 2 and 8, scaffolds containing large blocks of human X orthologs are interrupted by gene clusters corresponding to other human chromosomes (Additional file 1). Chromosome 2 contains nearly all the XAR genes (134), with 306 XCR orthologs found on chromosome 8 (Figure 2, Additional file 1). These results confirm the remarkable evolutionary conservation of chromosomal content noted in the genome assembly analysis [14], despite some bias due to easier identification of frog orthologs in synteny blocks where neighbouring gene identities are also conserved. The exceptions to the XCR and XAR conservation are two XCR genes found on chromosome 2 (scaffold 422). This is likely to result from a translocation in *Amphibia*, since chicken and opossum orthologs of these two genes reside as expected in chromosome 4 and X, respectively. The XAR–XCR boundary is located between the *RGN* and *PCTK1* genes, an interval containing the *NDUFB11RBM10* border previously suggested by human-marsupial comparisons [16]. The *X. tropicalis* sex determining locus has been mapped [17] to the neighbourhood of scaffolds 494, 605 and 735 on chromosome 7, and does not appear to be linked to amphibian X-borne genes.

### Is there a third evolutionary stratum in human X?

Comparisons of human X with the chicken genome have reached differing conclusions. The third evolutionary stratum on the human X identified by Kohn et al. [11] consists of the gene-rich regions Xp11 and Xq28, which were apparently conserved with chicken chromosome 12 and microchromosomes. However, these putative syntenic regions in the chicken genome have since been shown to consist of avian paralogs, with many true orthologs present in EST collections or unanchored contigs but missing from the chicken genome assembly [12], challenging the hypothesis of the third evolutionary stratum.

Our data strongly support a common ancient origin for the entire XCR. We identified 33 orthologs of Xq28 between *BGN* and *IKBKG* in the frog genome, of which 32 localize to chromosome 8 together with the remainder of the XCR (Additional file 1). Similarly, 21/22 Xp11 orthologs between *GPKOW* and *FAAH2* localized to frog chromosome 8. Between *RGN* and *GPKOW* in Xp11, synteny comparisons among amniotes have been problematic. While this region is well represented on the opossum X chromosome, data from other species comprise only six platypus genes from chromosome 6 and a mixture of orthologs and paralogs from chicken chromosomes 1, 12 and 4 [19]. We used FISH to obtain cytological locations for 13 orthologous frog genes from this area, with all placing to the short arm of chromosome 8.

Overall, we were able to locate putative frog orthologs of 68 human Xp11 and Xq28 genes. 66 of these are located on chromosome 8 together with the rest of the XCR orthology, indicating that the whole mammalian XCR shares ancestry with a single *X. tropicalis* chromosome. Delbridge et al. [12] also hypothesized that the Xp11 and Xq28 regions could have arisen from an ancient genome by segmental duplication, since paralogous regions exist on single autosomes in human, rat, opossum and chicken. Orthologs of human genes from both Xp11 and Xq28 were found together in the same frog scaffolds (154, 456, 507 and 690) as shown in Additional file 1. This is consistent with the Xp11 and Xq28 regions being located near each other deep in evolution, followed by segmental duplication before divergence of amniotes and amphibians.

### X chromosome deep evolution

Kohn et al. [11] have already suggested that XCR existed as an individual autosome in an amniote ancestor, because it persists as the single chromosome 4A in birds (except *Galliformes*) [20-22]. As mentioned above, this ancestral autosome likely acquired the sex-determining gene SRY after divergence of *Prototheria* and *Theria*, then fused with the XAR in the eutherian lineage. The distribution of human orthologs in frog chromosomes supports this single-chromosome origin for the XCR. In *X. tropicalis*, chromosome 8 contains not only XCR homology, but also homology to other human chromosomes (Figure 1). This suggests that in the amphibian lineage, the putative ancestral XCR fused with another autosome to form an initial frog chromosome 8, which was then reshaped by intrachromosomal rearrangements.

**Figure 3:**
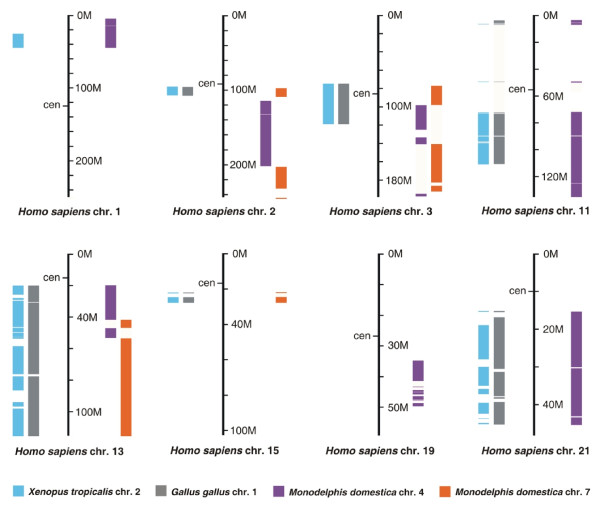
**Regions of human chromosomes homologous to frog, chicken and opossum chromosomes.** Opossum chromosomes 4 (orange) and 7 (purple) both show regions of homology on single human chromosomes 2,3, and 13, supporting their origin from a single ancestral chromosome. Amphibian (blue) and bird (grey). Figure summarizes data from Additional file 3. cen – centromere.

**Figure 4:**
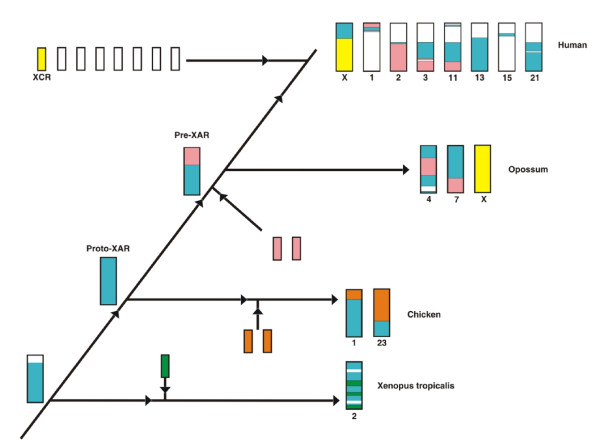
**Proposed origin of X-added region of human X chromosome.** A protochromosome (blue, left), present in the progenitor of *Synapsida * and *Sauropsida, * fused with several chromosomes (pink) to form the precursor of the eutherian X-Added Region (‘Pre-XAR’), now extant as most of opossum chromosomes 4 and 7. The Pre-XAR subsequently fragmented and fused with the therian X-Conserved Region (XAR and opossum X, yellow) to produce the eutherian X chromosome as well as with other partners (unshaded) contributing to autosomes. Fusion partners of the proto-XAR in amphibian (green) and avian (orange) lineages are also shown.

The history of the XAR is more complex. In all non-eutherian vertebrates studied, the regions corresponding to the XAR do not exist as separate cytological entities, but are present within chromosomes, surrounded by other conserved gene blocks that are autosomal in eutherians [8,14,23]. In order to trace the broader chromosomal context of XAR evolution, we examined homology of these nearby gene blocks in non-eutherian vertebrate genomes. Regions surrounding the identified XAR homology on opossum chromosomes 4 and 7, chicken chromosome 1, and frog chromosome 2 were compared to the human genome; incomplete genomic data for wallaby, platypus and the anole lizard preclude synteny analysis. Strikingly, these XAR-neighbouring regions of opossum chromosomes 4 and 7 showed coherent and complementary stretches of homology to parts of human chromosomes 2, 3, and 13 (Figure 3 and Additional file 3) previously hypothesized to derive from fission of a single predecessor [8,23]. The homology of these three human autosomes to both opossum 4 and 7 allows us to trace the genesis of the XAR in mammals. Localized human genome homology to both marsupial autosomes strongly supports a single pre-XAR chromosome, whose gene content was nearly identical to opossum chromosomes 4 and 7, which underwent a simple fission event to give these two autosomes in the marsupial lineage (Figure 4, second row). Human chromosomes 2, 3, and 13 show homology to both opossum chromosomes 4 and 7, and thus identify breakpoints in chromosomal rearrangement events following the divergence of marsupials from Eutheria. Human chromosomes with homology to either opossum chromosome 4 or 7, but not both (human chromosomes 11, 13, 15, and 21, Figure 3) are less informative since they do not evince breakpoints. The most parsimonious way to obtain the observed arrangement of homologies (including three breakpoints) in the eutherian lineage is a single internal translocation or inversion event in the pre-XAR, followed by fragmentation of the pre-XAR and fusion with XAR to form the eutherian X and autosomes (Figure 4, top row).

Our comparison of tetrapod genomes supports the following model for X evolution (Figure 4). The pre-XAR ancestral chromosome (see Figure 4, pink and blue second tier from top) can be defined as the sum of opossum chromosomes 4 and 7 (minus a region of chromosome 4 orthologous to human chromosome 19, which may represent a subsequent marsupial-specific fusion event). Differences in gene block order between human and opossum in this region suggest that in the period following divergence of marsupials but prior to the eutherian radiation, rearrangements inside the pre-XAR could have taken place. Further evolution of the eutherian karyotype then involves fragmentation of the pre-XAR chromosome, with the mature XAR joining the XCR (Figure 4, yellow) to complete the mammalian X, and the remaining pre-XAR fragments contributing to human chromosomes 1, 2, 3, 11, 13, 15 and 21.

Analysis of synteny data from frog and chicken genomes (Additional file 3) shows that deeper in evolution, regions corresponding to the pre-XAR share almost identical gene blocks with each other, but contain substantially fewer genes than the therian pre-XAR. We therefore infer the existence of a single proto-XAR chromosome, ancestral to the pre-XAR, in the progenitor of *Synapsida* and *Sauropsida* (Figure 4, blue). In the amphibian lineage, the proto-XAR region probably fused with another chromosome (Figure 4, green) to form frog chromosome 2. In birds, the proto-XAR now forms a major portion of chicken chromosome 1, plus a small region of chicken chromosome 23 (homologous to part of human chromosome 1 derived from the pre-XAR). Future availability of a more detailed anole genome may help identify differences in chromosomal evolution between birds and other lines of *Sauropsida*.

Fusion partners of the proto-XAR (Figure 4, pink), identified by their presence in opossum chromosomes, are found in chicken chromosomes 7, 9, 21 and 24 (Additional file 3). In therians, these fusions formed the pre-XAR, which then fragmented to give rise to large areas of human chromosomes 2, 3 and 11 homologous with opossum chromosomes 4 and 7. The structure of the human XAR differs from the corresponding part of the proto-XAR retained in frog by only a single translocation associated with inversion. Orthologs from frog scaffold 253 lie at the start and end of the XAR (Additional file 1), while their counterparts form a continuous region of chromosome 1 in chicken.

### Evolution of gene content

The human X chromosome is highly enriched for reproduction- and brain-related genes [24-26]. However, the human genome project detected negligible gene movement to the human X chromosome from autosomes [27], and brain-related genes on the human X and syntenic chicken chromosomes share an ancient origin [28]. Our analysis confirms minimal gene traffic from other chromosomes onto the mammalian X, as only 1.5% of human X chromosome single protein coding genes are found on *X. tropicalis* chromosomes other than chromosome 2 or 8, although identification of new frog orthologs could affect this ratio.

Segmental duplications resulting in tandemly arrayed genes (TAGs) are a source for emergence of new genes in mammalian and primate evolution [29-31]. Selection pressure sometimes results in repeated duplication of multigene segments. In yeast, the number of tandemly repeated units containing genes for metallothionein and an unrelated gene [32] increases or decreases via non reciprocal recombination in response to intensity of selection by copper. An intriguing convergent feature of human X and chicken Z chromosomes is the presence of TAGs with elevated expression in testis, while expression of single-copy conserved genes shows no sex bias [19]. These findings point to a central role for TAGs in evolution of human X chromosome gene content.

In the case of the human X chromosome, 5 of 15 tandemly-arrayed multigene families have single known orthologs in the *X. tropicalis* genome (Additional file 1). For example, the *MAGE* superfamily is represented by a single gene in *X. tropicalis* chromosome 8 [33]. The same frog chromosome bears single orthologs of the *SAGE1* and *CT45* families. The gene families *BEX, TCEAL (WEX), NXF* and *GPRASP (GASP)* evolved by gene conversion from a common ancestral *GPRASP*-like gene [34,35]. The frog genome contains a single ortholog (ENSXETG00000019743) of the *ARMCX-* and *GPRASP*-related gene family on chromosome 3, inferring the existence of a new superfamily located as a single block in human X. In addition to such single gene ancestors of amniote TAGs, ancient and conserved TAG clusters such as the ARSD family (Additional file 1) are also seen in the *X. tropicalis* genome.

## Conclusions

The comparison of amphibian and amniote genomes presented here traces the constituents of the human X chromosome back more than 300 MYA to the common ancestor of the tetrapod lineage. Chromosomal fusion partners and breakage events giving rise to the X-conserved and X-added regions and other domains can be inferred from extant genomic and cytogenetic evidence. This analysis demonstrates robust conservation of these chromosomal blocks and unambiguously confirms a 2-component model for the origin of the eutherian X chromosome.

## Methods

*Homo sapiens* Genome Build 37.1 [36] served as the source of the human gene list. We excluded pseudogenes, gene models, microRNAs and miscellaneous RNAs from the evaluation, leaving 182 genes in the human XAR and 627 from the XCR. *X. tropicalis* orthologs were then identified individually in Ensembl [37] and Xenbase [38] databases. Xenbase, the principle source of *X.tropicalis* orthologs, contains 4705 manually-annotated and 10,833 machine-annotated gene pages. Entries on the gene list were based on e-values of 1e^-10^ with a minimum 55% identity and 65% coverage [39]. Chromosomal locations of some scaffolds (JGI *X. tropicalis* genome assembly 4.1) containing identified orthologs were obtained from the existing *X. tropicalis* linkage map [15]. Information about blocks that are homologous between human, opossum and chicken (Additional file 3) is from the Comparative Genomics display of the Ensembl database [18]. Orthologs of human genes in gaps between synteny blocks were identified in databases [37,38] and the *X. tropicalis* linkage map [15]. Some families of human duplicated genes have single known ancestral orthologs, which were only counted once. In total, we were able to identify chromosomal locations in the *X. tropicalis* genome for 454 human X orthologs.

For certain *X. tropicalis* sequence scaffolds not represented on the current linkage map, we also obtained cytological locations using fluorescent in situ hybridization coupled with tyramide amplification (FISH-TSA) using chosen scaffold-specific cDNA probes [40,41]. Probes for chromosomal in situ hybridization were generated from cDNAs of frog orthologs of human X chromosome genes described in Additional file 4. Images of chromosomes visualized with the fluorophores tetramethylrhodamine and diamidinophenylindole were collected at two different wavelengths (U-MWV and U-MWIY filters) on an Olympus BX40 microscope with 100x objective using a Sony SPT-M320CE camera. Contrast and brightness were adjusted using the ACC program (Sofo, Brno) and the images merged in pseudocolor.

## Abbreviations

*X. tropicalis*: *Xenopus tropicalis*; XAR: X chromosome added region; XCR: X chromosome conserved region; FISH-TSA: Fluorescent in situ hybridization coupled with tyramide amplification; pre-XAR: Ancestral pre-XAR chromosome in therian mammals; proto-XAR: Predecessor of X chromosome added region in Tetrapoda; TAG: Tandemly arrayed genes.

## Competing interests

The authors declare no competing financial interests. Correspondence and requests for materials should be addressed to lzimmer@nimr.mrc.ac.uk.

## Authors’ contributions

JM, RT, AS, DW, and VK collected and analysed data, RT and TT performed gene visualisation and JM, LZ and RT wrote the paper. All authors read and approved the final manuscript.

## Supplementary Material

Additional file 1 **Human X-borne genes and their *X. tropicalis *orthologs. ** Table lists genes from *Homo sapiens * Genome Build 37.1 [36] and their *X. tropicalis * orthologs obtained from Ensembl [37] and Xenbase [38] databases. Human tandemly-arrayed genes are highlighted in yellow, *X. tropicalis * orthologs on chromosome 2 are highlighted in blue, *X. tropicalis * orthologs on chromosome 8 are highlighted in green, *X. tropicalis * orthologs in other chromosomes are highlighted in red, and the border gene between XCR and XAR is highlighted in grey. Click here for file

Additional file 2 **Cytogenetic localisation of*X. tropicalis*genes in chromosomes.** FISH-TSA localization of genes from reference scaffolds onto *X. tropicalis* chromosomes using scaffold-specific cDNA probes. Probes are described in Additional file 4. Click here for file

Additional file 3 **Chromosomal positions of orthologs of genes from human chromosomes 1, 2, 3, 11, 13, 15, 19 and 21.** Table shows position of human gene orthologs in *X. tropicalis, * chicken and opossum chromosomes. Syntenic blocks between human, opossum and chicken are from the Comparative Genomics display of the Ensembl database [18]. Orthologs of human genes in gaps between synteny blocks were identified in databases [37,38] and the *X. tropicalis * linkage map [15]. Click here for file

Additional file 4 **Probes used for gene visualization by FISH-TSA.** Table contains corresponding scaffolds and description of cDNA probes for FISH-TSA. Click here for file

## References

[B1] Bourque G Zdobnov EM Bork P Pevzner PA Tesler G Comparative architectures of mammalian and chicken genomes reveal highly variable rates of genomic rearrangements across different lineages Genome Res 2005 15 98 110 10.1101/gr.3002305 15590940 PMC540283

[B2] Murphy WJ Larkin DM Everts-van der Wind A Bourque G Tesler G Auvil L Beever JE Chowdhary BP Galibert F Gatzke L Hitte C Meyers SN Milan D Ostrander EA Pape G Parker HG Raudsepp T Rogatcheva MB Schook LB Skow LC Welge M Womack JE O'brien SJ Pevzner PA Lewin HA Dynamics of mammalian chromosome evolution inferred from multispecies comparative maps Science 2005 309 613 617 10.1126/science.1111387 16040707

[B3] Ma J Zhang L Suh BB Raney BJ Burhans RC Kent WJ Blanchette M Haussler D Miller W Reconstructing contiguous regions of an ancestral genome Genome Res 2006 16 1557 1565 10.1101/gr.5383506 16983148 PMC1665639

[B4] Jaillon O Aury JM Brunet F Petit JL Stange-Thomann N Mauceli E Bouneau L Fischer C Ozouf-Costaz C Bernot A Nicaud S Jaffe D Fisher S Lutfalla G Dossat C Segurens B Dasilva C Salanoubat M Levy M Boudet N Castellano S Anthouard V Jubin C Castelli V Katinka M Vacherie B Biémont C Skalli Z Cattolico L Poulain J Genome duplication in the teleost fish Tetraodon nigroviridis reveals the early vertebrate proto-karyotype Nature 2004 431 946 957 10.1038/nature03025 15496914

[B5] Graves JA The origin and function of the mammalian Y chromosome and Y-borne genes–an evolving understanding Bioessays 1995 17 311 320 10.1002/bies.950170407 7741724

[B6] Bininda-Emonds OR Cardillo M Jones KE MacPhee RD Beck RM Grenyer R Price SA Vos RA Gittleman JL Purvis A The delayed rise of present-day mammals Nature 2007 446 507 512 10.1038/nature05634 17392779

[B7] Warren WC Hillier LW Marshall Graves JA Birney E Ponting CP Grützner F Belov K Miller W Clarke L Chinwalla AT Yang SP Heger A Locke DP Miethke P Waters PD Veyrunes F Fulton L Fulton B Graves T Wallis J Puente XS López-Otín C Ordóñez GR Eichler EE Chen L Cheng Z Deakin JE Alsop A Thompson K Kirby P Genome analysis of the platypus reveals unique signatures of evolution Nature 2008 453 175 183 10.1038/nature06936 18464734 PMC2803040

[B8] Veyrunes F Waters PD Miethke P Rens W McMillan D Alsop AE Grützner F Deakin JE Whittington CM Schatzkamer K Kremitzki CL Graves T Ferguson-Smith MA Warren W Marshall Graves JA Bird-like sex chromosomes of platypus imply recent origin of mammal sex chromosomes Genome Res 2008 18 965 973 10.1101/gr.7101908 18463302 PMC2413164

[B9] Wallis MC Delbridge ML Pask AJ Alsop AE Grutzner F O'Brien PC Rens W Ferguson-Smith MA Graves JA Mapping platypus SOX genes; autosomal location of SOX9 excludes it from sex determining role Cytogenet Genome Res 2007 116 232 234 10.1159/000098192 17317965

[B10] Ross MT Grafham DV Coffey AJ Scherer S McLay K Muzny D Platzer M Howell GR Burrows C Bird CP Frankish A Lovell FL Howe KL Ashurst JL Fulton RS Sudbrak R Wen G Jones MC Hurles ME Andrews TD Scott CE Searle S Ramser J Whittaker A Deadman R Carter NP Hunt SE Chen R Cree A Gunaratne P The DNA sequence of the human X chromosome Nature 2005 434 325 337 10.1038/nature03440 15772651 PMC2665286

[B11] Kohn M Kehrer-Sawatzki H Vogel W Graves JA Hameister H Wide genome comparisons reveal the origins of the human X chromosome Trends Genet 2004 20 598 603 10.1016/j.tig.2004.09.008 15522454

[B12] Delbridge ML Patel HR Waters PD McMillan DA Marshall Graves JA Does the human X contain a third evolutionary block? Origin of genes on human Xp11 and Xq28 Genome Res 2009 19 1350 1360 10.1101/gr.088625.108 19439513 PMC2720175

[B13] Ravi V Venkatesh B Rapidly evolving fish genomes and teleost diversity Curr Opin Genet Dev 2008 18 544 550 10.1016/j.gde.2008.11.001 19095434

[B14] Hellsten U Harland RM Gilchrist MJ Hendrix D Jurka J Kapitonov V Ovcharenko I Putnam NH Shu S Taher L Blitz IL Blumberg B Dichmann DS Dubchak I Amaya E Detter JC Fletcher R Gerhard DS Goodstein D Graves T Grigoriev IV Grimwood J Kawashima T Lindquist E Lucas SM Mead PE Mitros T Ogino H Ohta Y Poliakov AV The genome of the Western clawed frog Xenopus tropicalis Science 2010 328 633 636 10.1126/science.1183670 20431018 PMC2994648

[B15] Wells DE Gutierrez L Xu Z Krylov V Macha J Blankenburg KP Hitchens M Bellot LJ Spivey M Stemple DL Kowis A Ye Y Pasternak S Owen J Tran T Slavikova R Tumova L Tlapakova T Seifertova E Scherer SE Sater AK A genetic map of Xenopus tropicalis Dev Biol 2011 354 1 8 10.1016/j.ydbio.2011.03.022 21458440 PMC3098391

[B16] Mikkelsen TS Wakefield MJ Aken B Amemiya CT Chang JL Duke S Garber M Gentles AJ Goodstadt L Heger A Jurka J Kamal M Mauceli E Searle SM Sharpe T Baker ML Batzer MA Benos PV Belov K Clamp M Cook A Cuff J Das R Davidow L Deakin JE Fazzari MJ Glass JL Grabherr M Greally JM Gu W Genome of the marsupial Monodelphis domestica reveals innovation in non-coding sequences Nature 2007 447 167 177 10.1038/nature05805 17495919

[B17] Olmstead AW Lindberg-Livingston A Degitz SJ Genotyping sex in the amphibian, Xenopus (Silurana) tropicalis, for endocrine disruptor bioassays Aquat Toxicol 2010 98 60 66 10.1016/j.aquatox.2010.01.012 20202696

[B18] The Comparative Genomics display of the Ensembl database http://www.ensembl.org/Homo_sapiens/Location/Synteny?r=X

[B19] Bellott DW Skaletsky H Pyntikova T Mardis ER Graves T Kremitzki C Brown LG Rozen S Warren WC Wilson RK Page DC Convergent evolution of chicken Z and human X chromosomes by expansion and gene acquisition Nature 2010 466 612 616 10.1038/nature09172 20622855 PMC2943333

[B20] Shibusawa M Nishida-Umehara C Masabanda J Griffin DK Isobe T Matsuda Y Chromosome rearrangements between chicken and guinea fowl defined by comparative chromosome painting and FISH mapping of DNA clones Cytogenet Genome Res 2002 98 225 230 10.1159/000069813 12698009

[B21] Guttenbach M Nanda I Feichtinger W Masabanda JS Griffin DK Schmid M Comparative chromosome painting of chicken autosomal paints 1–9 in nine different bird species Cytogenet Genome Res 2003 103 173 184 10.1159/000076309 15004483

[B22] Stapley J Birkhead TR Burke T Slate J A linkage map of the zebra finch Taeniopygia guttata provides new insights into avian genome evolution Genetics 2008 179 651 667 10.1534/genetics.107.086264 18493078 PMC2390641

[B23] Deakin JE Koina E Waters PD Doherty R Patel VS Delbridge ML Dobson B Fong J Hu Y van den Hurk C Pask AJ Shaw G Smith C Thompson K Wakefield MJ Yu H Renfree MB Graves JA Physical map of two tammar wallaby chromosomes: a strategy for mapping in non-model mammals Chromosome Res 2008 16 1159 1175 10.1007/s10577-008-1266-y 18987984

[B24] Saifi GM Chandra HS An apparent excess of sex- and reproduction-related genes on the human X chromosome Proc Biol Sci 1999 266 203 209 10.1098/rspb.1999.0623 10097393 PMC1689664

[B25] Chelly J Mandel JL Monogenic causes of X-linked mental retardation Nat Rev Genet 2001 2 669 680 11533716 10.1038/35088558

[B26] Zechner U Wilda M Kehrer-Sawatzki H Vogel W Fundele R Hameister H A high density of X-linked genes for general cognitive ability: a run-away process shaping human evolution? Trends Genet 2001 17 697 701 10.1016/S0168-9525(01)02446-5 11718922

[B27] Venter JC Adams MD Myers EW Li PW Mural RJ Sutton GG Smith HO Yandell M Evans CA Holt RA Gocayne JD Amanatides P Ballew RM Huson DH Wortman JR Zhang Q Kodira CD Zheng XH Chen L Skupski M Subramanian G Thomas PD Zhang J Gabor Miklos GL Nelson C Broder S Clark AG Nadeau J McKusick VA Zinder N The sequence of the human genome Science 2001 291 1304 1351 10.1126/science.1058040 11181995

[B28] Kemkemer C Kohn M Kehrer-Sawatzki H Fundele RH Hameister H Enrichment of brain-related genes on the mammalian X chromosome is ancient and predates the divergence of synapsid and sauropsid lineages Chromosome Res 2009 17 811 820 10.1007/s10577-009-9072-8 19731051

[B29] Bailey JA Eichler EE Primate segmental duplications: crucibles of evolution, diversity and disease Nat Rev Genet 2006 7 552 564 16770338 10.1038/nrg1895

[B30] Liu GE Ventura M Cellamare A Chen L Cheng Z Zhu B Li C Song J Eichler EE Analysis of recent segmental duplications in the bovine genome BMC Genomics 2009 10 571 10.1186/1471-2164-10-571 19951423 PMC2796684

[B31] Marques-Bonet T Girirajan S Eichler EE The origins and impact of primate segmental duplications Trends Genet 2009 25 443 544 10.1016/j.tig.2009.08.002 19796838 PMC2847396

[B32] Welch J Fogel S Buchman C Karin M The CUP2 gene product regulates the expression of the CUP1 gene, coding for yeast metallothionein EMBO J 1989 8 255 260 2653812 10.1002/j.1460-2075.1989.tb03371.x PMC400797

[B33] Lopez-Sanchez N Gonzalez-Fernandez Z Niinobe M Yoshikawa K Frade JM Single mage gene in the chicken genome encodes CMage, a protein with functional similarities to mammalian type II Mage proteins Physiol Genomics 2007 30 156 171 10.1152/physiolgenomics.00249.2006 17374844

[B34] Winter EE Ponting CP Mammalian BEX, WEX and GASP genes: coding and non-coding chimaerism sustained by gene conversion events BMC Evol Biol 2005 5 54 10.1186/1471-2148-5-54 16221301 PMC1274310

[B35] Zhang L Adaptive evolution and frequent gene conversion in the brain expressed X-linked gene family in mammals Biochem Genet 2008 46 293 311 10.1007/s10528-008-9148-8 18236150

[B36] Homo sapiens Genome Build 37.1 http://www.ncbi.nlm.nih.gov/projects/mapview/map_search.cgi?taxid=9606

[B37] Ensembl: Genome databases for vertebrates and other eukaryotic specieshttp://www.ensembl.org/index.html

[B38] Xenbase: Xenopus laevis and Xenopus tropicalis biology and genomics resourcehttp://www.xenbase.org/common/10.1093/nar/gkm826 PMC2238855 17984085

[B39] Bowes JB Snyder KA Segerdell E Jarabek CJ Azam K Zorn AM Vize PD Xenbase: gene expression and improved integration Nucleic Acids Res 2010 38 D607 D612 10.1093/nar/gkp953 19884130 PMC2808955

[B40] Khokha MK Krylov V Reilly MJ Gall JG Bhattacharya D Cheung CY Kaufman S Lam DK Macha J Ngo C Prakash N Schmidt P Tlapakova T Trivedi T Tumova L Abu-Daya A Geach T Vendrell E Ironfield H Sinzelle L Sater AK Wells DE Harland RM Zimmerman LB Rapid gynogenetic mapping of Xenopus tropicalis mutations to chromosomes Dev Dyn 2009 238 1398 1406 10.1002/dvdy.21965 19441086 PMC2962985

[B41] Tlapakova T Krylov V Macha J Localization, structure and polymorphism of two paralogous Xenopus laevis mitochondrial malate dehydrogenase genes Chromosome Res 2005 13 699 706 10.1007/s10577-005-0987-4 16235119

