# Supplementary figures and images for: Deep ancestry of mammalian X chromosome revealed by comparison with the basal tetrapod Xenopus tropicalis
Source: BMC Genomics. 2012 Jul 16;13:315. doi: 10.1186/1471-2164-13-315 (PMC3472169; doi:10.1186/1471-2164-13-315)

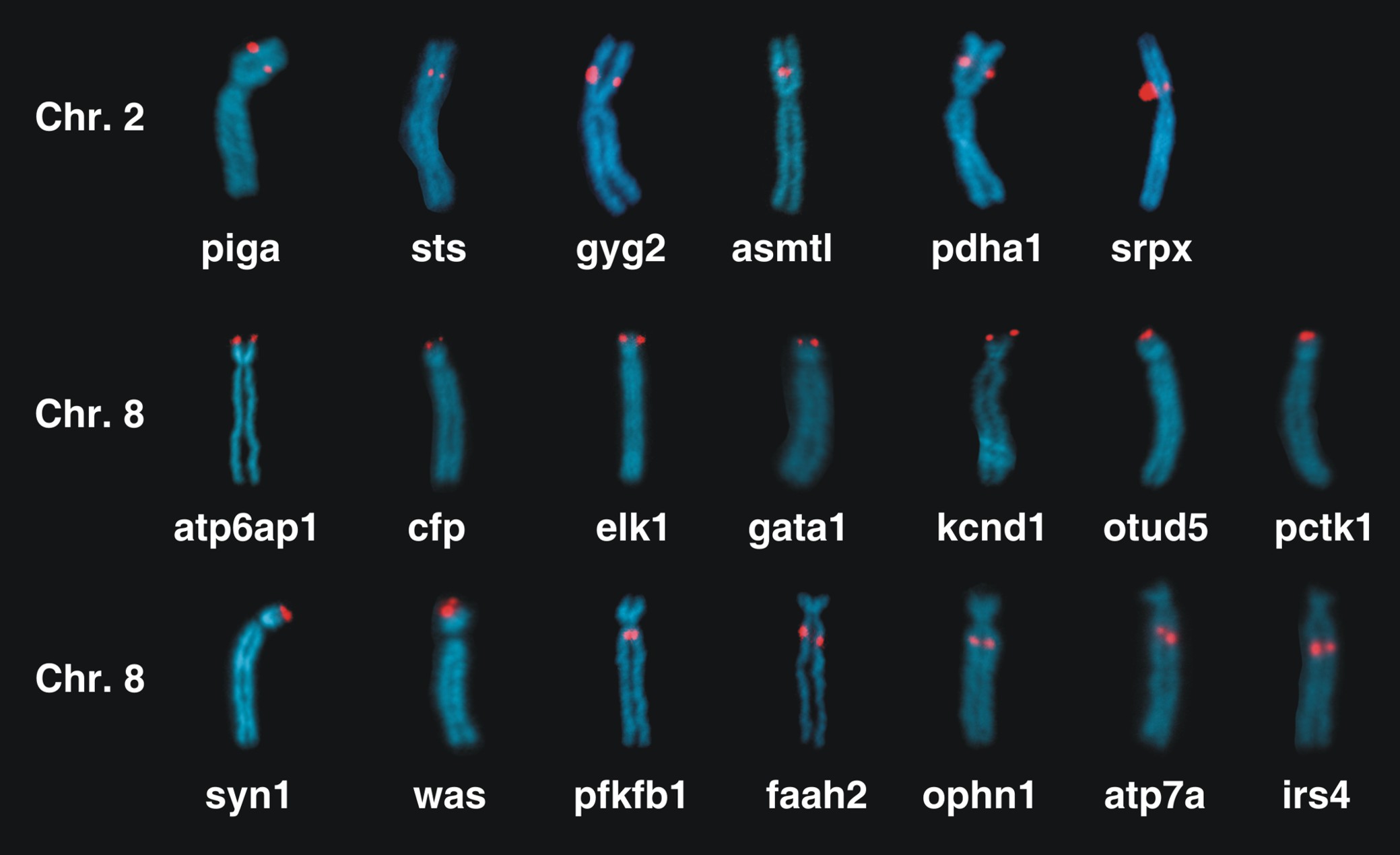

Supplement: Additional file 2 — Cytogenetic localisation ofX. tropicalisgenes in chromosomes. FISH-TSA localization of genes from reference scaffolds onto X. tropicalis chromosomes using scaffold-specific cDNA probes. Probes are described in Additional file 4. [file 1471-2164-13-315-S2.jpeg]
